# Real-world outcomes of an innovative digital therapeutic for treatment of panic disorder and PTSD: A 1,500 patient effectiveness study

**DOI:** 10.3389/fdgth.2022.976001

**Published:** 2022-11-17

**Authors:** Robert N. Cuyler, Rahul Katdare, Simon Thomas, Michael J. Telch

**Affiliations:** ^1^ Freespira, Inc., Houston, TX, United States; ^2^ Freespira, Inc., Kirkland, WA, United States; ^3^ Laboratory for the Study of Anxiety Disorders, University of Texas, Austin, TX, United States

**Keywords:** CGRI, panic disorder, PTSD, digital therapeutic, telehealth, biofeedback, carbon dioxide hypersensitivity

## Abstract

**Objective:**

Prior clinical trials have shown consistent clinical benefit for Capnometry Guided Respiratory Intervention (CGRI), a prescription digital therapeutic for the treatment of panic disorder (PD) and post-traumatic stress disorder (PTSD). The purpose of this study is to report real-world outcomes in a series of patients treated with the intervention in clinical practice.

**Design:**

This paper reports pre- and post-treatment self-reported symptom reduction, measures of respiratory rate and end-tidal carbon dioxide levels, drop-out and adherence rates drawn from an automatic data repository in a large real-world series of patients receiving CGRI for panic disorder and PTSD.

**Setting:**

Patients used the intervention in their homes, supported by telehealth coaching.

**Participants:**

Patients meeting symptom criteria for panic disorder (*n* = 1,395) or posttraumatic stress disorder (*n* = 174) were treated following assessment by a healthcare professional.

**Intervention:**

Capnometry Guided Respiratory Intervention is a 28-day home-based treatment that provides breath-to-breath feedback of respiratory rate and exhaled carbon dioxide levels, aimed at normalizing respiratory style and increasing patients’ mastery for coping with symptoms of stress, anxiety, and panic. Health coaches provide initial training with weekly follow up during the treatment episode. Remote data upload and monitoring facilitates individualized coaching and aggregate outcomes analysis.

**Main outcome measures:**

Self-reported Panic Disorder Severity Scale (PDSS) and the Posttraumatic Stress Disorder Checklist for DSM-5 (PCL-5) scores were obtained at pre-treatment and post-treatment.

**Results:**

Panic disorder (PD) patients showed a mean pre-to-post-treatment reduction in total PDSS scores of 50.2% (*P* < 0.001, *d* = 1.31). Treatment response rates for PD (defined as a 40% or greater reduction in PDSS total scores) were observed in 65.3% of the PD patients. PTSD patients showed a pre-to-post-treatment reduction in total PCL-5 scores of 41.1% (*P* < 0.001, *d* = 1.16). The treatment response rate for PTSD (defined as a ≥10-point reduction in PCL-5 scores) was 72.4%. In an additional analysis of response at the individual level, 55.7% of panic disorder patients and 53.5% of PTSD patients were classified as treatment responders using the Reliable Change Index. Patients with both normal and below-normal baseline exhaled CO_2_ levels experienced comparable benefit. Across the 28-day treatment period, mean adherence rates of 74.8% (PD) and 74.9% (PTSD) were recorded during the 28-day treatment. Dropout rates were 10% (PD) and 11% (PTSD) respectively.

**Conclusions:**

The results from this cohort of 1,569 patients treated with the CGRI intervention demonstrate significant rates of symptom reduction and adherence consistent with prior published clinical trials. The brief duration of treatment, high adherence rates, and clinical benefit suggests that CGRI provides an important addition to treatment options for panic disorder and PTSD.

## Introduction

Panic disorder (PD) and Post-traumatic Stress Disorder (PTSD) are common and often become chronic behavioral health conditions. Lifetime prevalence for isolated panic attacks is reported at 22.7% and lifetime prevalence for full criteria panic disorder is estimated at 4.8% (1). Estimates of lifetime PTSD prevalence range from 3.4% to 8.0% in the general population and 7.7% to 13.4% in veterans (2). The most widely utilized and recommended current treatments are psychotropic medications and/or psychotherapy. In the case of panic disorder and recurrent panic symptoms, review of pharmacologic treatments shows substantial rates of inadequate response, with many patients experiencing chronic relapsing conditions (3). When the nature of actual care delivered in routine clinical practice rather than published trials is examined, most patients with panic symptoms are seen in the general medical sector and the majority of patients do not receive evidence-based pharmacologic or psychotherapeutic treatments (1). Similarly, PTSD often takes a chronic course with up to a third of individuals remaining symptomatic a decade after trauma exposure (4). A meta-analysis of placebo-controlled studies of cognitive behavioral therapies show small to medium effect sizes for PD and PTSD as compared to more robust results for OCD, social anxiety disorder, and acute stress disorder (5).

A common limitation of psychotherapeutic and pharmacologic approaches to PD and PTSD is that neither address the role of respiratory physiology and breathing style. A very useful review by Boulding and colleagues (6) examines relevant literature and proposes a useful classification of respiratory styles (termed “dysfunctional breathing”) implicated in a spectrum of health conditions including panic. More specifically, a substantial body of work posits situational as well as chronic dysfunctional breathing as risk factors in panic attacks and the subsequent development of panic disorder (7). Evidence supporting the respiratory dysregulation hypothesis comes from a substantial body of work linking CO_2_ hypersensitivity to panic attacks and panic disorder, initiated in large part by Klein’s conceptualization of a faulty suffocation alarm (8, 9). An important “marker” of this hypersensitivity comes from studies of carbon dioxide challenge testing. In experimental lab settings, researchers have established that compared to healthy controls, most panic sufferers (and many close relatives) react with pronounced panic symptoms, including fear and physiological distress, when exposed to single or repeated breaths of CO_2_- enriched air (10–15).

A smaller body of work has identified similar reactivity for individuals with PTSD. A double-blind, randomized control study of reactivity to CO_2_ challenge showed that diagnosed PTSD patients were highly reactive to inhaled 35% CO_2_ but not to a placebo gas mixture, while healthy controls were largely unaffected (16). In addition, soldiers who demonstrated high distress during CO_2_ challenge were found to be at higher risk than non-reactors for developing PTSD symptoms during deployment to Iraq (17).

This evidence of CO_2_ hypersensitivity as a common risk factor for both panic and PTSD, as well as evidence of a bidirectional relationship between the two conditions (18), provided a compelling rationale that led our treatment development team to develop Freespira®, a digital therapeutic specifically targeting normalization of dysfunctional breathing patterns *via* Capnometry-Guided Respiratory Intervention (CGRI); the intervention received FDA-clearance for treatment of panic disorder in 2013 and in 2018 for PTSD.

### Origins and previous efficacy trials

Research conducted by Meuret and colleagues (19) established a treatment protocol (Capnometry Assisted Respiratory Therapy, or CART) using feedback of respiratory rate and exhaled CO_2_. This trial showed significant and sustained symptomatic improvement in panic disorder severity with reported 93% treatment response (≥40% reduction in Panic Disorder Severity Scale (PDSS) scores) one-year post-treatment and large effect size (Cohen’s *d* = 2.6). This trial used commercially available capnometers and cassette-tape-recorded pacing tones for delivery of the CART protocol.

The CGRI intervention described in this paper represents an adaptation of the core Meuret protocol, using different instrumentation as well as imbedded data capture and remote review capabilities. A multi-center benchmarking study (20) offering CGRI in four independent anxiety treatment centers showed one-year response rates (≥40% reduction in PDSS scores) of 82% in treatment completers and large effect size (Cohen’s *d* = 2.3). Additionally, the authors identified subsets of participants classified as hypocapnic or normocapnic based on baseline averages of etCO_2_. Hypocapnic subjects experienced greater increases in exhaled carbon dioxide levels at post-treatment but the authors determined that the intervention produced equivalent clinical benefit post-treatment for normocapnic as well as hypocapnic subjects.

A health economic outcome study (21) undertaken by Highmark Health and Allegheny Health Network reported 91% response rates and 68% remission rates (PDSS scores ≤5) one-year post-CGRI treatment. Highmark, the insurer of the participant patients, compiled cost data by comparing paid healthcare claims (all sources) for the one year prior to and one year following the 28-day treatment. The study reported a 35% reduction in overall paid claims, a 65% reduction in emergency department costs, and a 68% reduction in pharmaceutical costs for the study participants.

A real-world study (22) was conducted in an employer-sponsored health clinic. CGRI was offered to patients seeking treatment for panic-related symptoms, following identification by primary care or behavioral health staff clinicians. In this case series, 18 participants with panic showed mean PDSS decreases of 7.2 scale points, with 67% showing significant reductions in PDSS scores (≥40%). Participants additionally showed decreases in behavioral health visits post-intervention.

An open label clinical trial (23) offering CGRI for treatment of PTSD was conducted at the Palo Alto VA, with enrollment open to both veterans and civilians. Mean Clinician-Administered PTSD Scale for DSM-5 (CAPS-5) scores declined significantly from pre- to post-treatment (49.5 to 27.1; Cohen’s *d* = 1.3). Moreover, at six-month follow-up, 50% were rated as “in remission”(based on post-treatment CAPS-5 showing significant reduction from baseline, an absolute score ≤ 25, plus no longer meeting DSM-5 criteria for PTSD as rated by a study clinician). Treatment completers averaged 77% adherence (i.e., completion of 43 of 56 recommended sessions). Similar to the prior PD trial (20), hypocapnic subjects significantly increased etCO_2_ levels from pre- to post-treatment. Both hypocapnic and normocapnic cohorts experienced significant reductions in CAPS-5 scores at six-month follow up, with a larger effect size (2.3) for the hypocapnic group compared to the normocapnic group (0.8).

### Proposed mechanism(s) of action

Several potential mechanisms of action have been proposed for the CART/CGRI protocols. As noted above (21, 23), symptomatic improvement in both panic and PTSD is associated with the ability of hypocapnic users to normalize etCO_2_ levels. Meuret et al. (24), in a randomized trial comparing CART with a cognitive therapy, found evidence of equivalent effectiveness but specific benefit in normalizing CO_2_ in the CART group while identifying perceived control (but not increase in etCO_2_) as a putative mechanism in the cognitive condition. Meuret and colleagues (25) additionally suggested that repeated exposure to respiratory distress may have led to an attenuation of respiratory distress *via* induction of dyspnea during the treatment.

Feinstein (26) and colleagues in a recent paper provide elegant synthesis and conceptualization of CO_2_ hypersensitivity, neuroanatomy, acid-base balance, and the role of chemoreceptors in anxiety conditions. The authors introduce the concept of “apnea induced anxiety”, which they interpret as a “an evolutionarily determined manifestation of the broader freezing response that the amygdala is well-known to coordinate”. One implication of the Feinstein paper is the possibility that, in addition to the role of desensitization and development of enhanced sense of control and self-efficacy described above, the CART/CGRI intervention may also function to inhibit abrupt, de-stabilizing spikes in CO_2_ and pH (27) provoked by dysfunctional breathing that induce anxiety and avoidance behaviors.

### Study rationale

This paper reports treatment effectiveness data on patients treated with CGRI in clinical practice. The large pool of completed treatments available here represents an opportunity to evaluate real world effectiveness of CGRI as a follow-up to the prior clinical trials.

## Materials and methods

### Treatment device and protocol

CGRI teaches a specific breathing style *via* a system providing real-time feedback of respiratory rate (RR) and exhaled carbon dioxide (etCO_2_) levels facilitated by health coaching and data capture. The CGRI system described in this paper combines: (a) a proprietary sensor for measurement of respiratory data, (b) an app-based respiratory feedback protocol pre-loaded on a tablet running Android 4.0 or higher (c) secure automatic data capture of adherence, physiological, and symptom severity metrics, and (d) telehealth training/coaching to educate and support patient use of the system.

**Figure 1:**
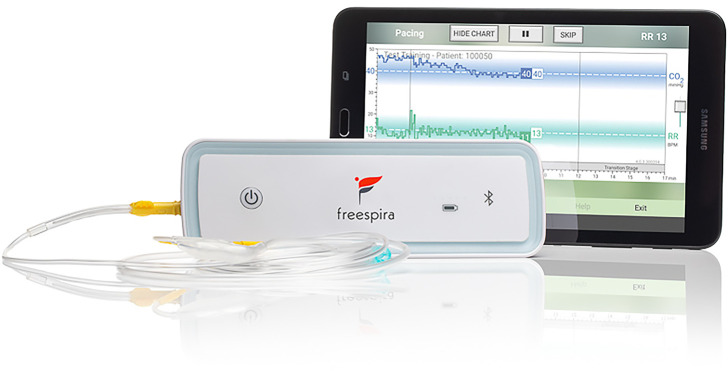
CGRI system.

The respiratory sensor (see Figure 1) measures breath-to-breath RR and etCO_2_ sampled *via* a small diameter nasal cannula. During a treatment session, real-time physiologic parameters are calculated by the sensor and transmitted to the Bluetooth®-connected tablet running dedicated, proprietary software. Respiratory data are graphically and numerically displayed, and audio/text instructions are provided by the tablet app. Per FDA clearance, prospective patients are authorized for the treatment by a licensed healthcare provider who affirms presence of the relevant conditions (panic disorder, panic attacks, or PTSD) and absence of contra-indications such as pregnancy, severe COPD or unstable psychiatric condition.

**Figure 2:**
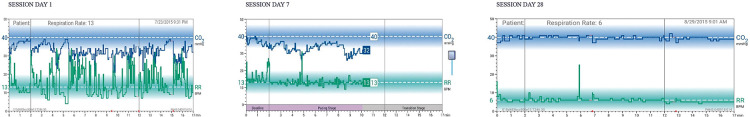
Respiratory feedback display.

Twice-daily sessions for 28 days are recommended. Each 17-min session comprises three stages: (a) two minutes of ***baseline*** respiratory measurement (patients are instructed to breathe as usual with eyes closed), (b) 10 min of respiratory ***pacing*** measurement (patients are instructed to breathe in sync with a rising and falling audio tone and to adjust respiratory volume guided by display of etCO_2_ levels relative to the normal range, and (c) five minutes of ***transition*** measurement (maintain paced breathing and etCO_2_ level without cueing by audio tones). The rationale of this final phase is to “stamp in” the targeted respiratory style with reduced feedback, thus engendering self-management skills that promote awareness of the onset of dysregulated breathing and the capacity to substitute the learned breathing style. An actual patient example of RR and etCO_2_ graphs at baseline, 7 days, and 28 days is seen in Figure 2. Note the progressive normalization of etCO_2_ values and slowing/stabilization of respiratory rate during the 28-day course of treatment.

Patients are classified at the initiation of treatment as panic or PTSD based on their initial clinical assessment, with all patients receiving an identical treatment protocol. Patients complete a baseline Panic Disorder Severity Scale (PDSS) (28) or PTSD Checklist for DSM-5 (PCL-5) (29) followed by self-report PDSS measurements (panic patients) or PCL-5 at post-treatment *via* embedded scale questions on the tablet computer.

An initial 45-min secure video teleconference is conducted with an assigned health coach who provides: (a) the treatment rationale, (b) determination of patient goals/expectations, (c) education regarding diaphragmatic breathing and respiratory targets, (d) instructions for using the sensor/tablet, and (e) observation/feedback while the patient undertakes an initial session. Weekly 10–15-min follow-up sessions with the health coach review the prior week’s sessions (available to the patient on the tablet and the coach on a secure portal) and provide coaching for continued progress. Although interactive video is the preferred method for these follow-up sessions, communication *via* phone or text can be substituted based on patient preference and progress. Weekly coaching notes are reviewed by clinical management and an end-of-treatment summary report consisting of initial and final session graphs, adherence information, coach observations, and symptom changes during treatment is sent to collaborating clinicians.

### Remote monitoring and data capture

Following each session, data are uploaded from the tablet to a secure server. Data include breath-by-breath physiological data (mean respiratory rates and mean etCO_2_ values for each of the session stages), self-reported symptoms as indexed by the PDSS or PCL-5 surveys, and images of the session graph (identical to what was seen by the patient on the tablet). Uploaded session data are maintained in a database with query and viewing tools facilitating longitudinal review of sessions by the assigned health coach. This review provides valuable information for identifying and addressing issues related to adherence problems, difficulties attaining respiratory targets, and symptom changes. Aggregation of patient data on a monthly, quarterly, and annual basis are conducted to track overall adherence, patient attrition, and clinical response rates.

## Methods

### Participants and source

This sample is comprised of 1,569 patients treated with CGRI between September 01, 2017 and September 16, 2021, drawn from a larger pool of 3,050 total patients treated with the intervention since first availability. Participant demographics are seen in Table 1. As consumers of routine clinical care, patients included insured and self-pay participants and were not paid for participation in this study. Self-report clinical rating surveys were not embedded into the tablet software until September 2017. Therefore, the 1,481 patients treated prior to availability of the in-app survey are excluded from this analysis. These 1,569 patients with pre/post surveys represent 89% of treatment completers during the study period, meaning that completed end-of-treatment surveys were missing for 11% of patients. During the study time frame, 10% (279) of PD patients and 11% (36) of PTSD patients were classified as dropouts, having completed fewer than 6 total sessions. Reasons for drop out were not systematically recorded.

**Table 1:** Demographic breakdown by diagnostic group.

	PD (*N* = 1,395)		PTSD (*N* = 174)	
	*M*	SD	*M*	SD
Age	39.2	13.9	40.9	14.9
Gender	*N*	%	*N*	%
Women	1,060	76	127	73
Men	335	24	46	26
Unknown	0	0	1	1

As per FDA requirements, patients were authorized for treatment by a licensed healthcare provider. Authorizing clinicians included both independent practicing professionals as well as contracted, state-licensed professionals who obtained a health history, confirmed the absence of contraindications, and obtained a pre-treatment PDSS or PCL-5 to determine eligibility and baseline symptom severity. Individuals who were under the age of 13, pregnant, had COPD or other advanced respiratory illness, inadequately controlled seizures or asthma, active suicidal ideation, schizophrenia, or active psychosis were screened out. Authorizing clinicians required individuals with medical complexity or Covid-19 history and residual respiratory symptoms to obtain additional medical clearance from a personal physician.

**Figure 3:**
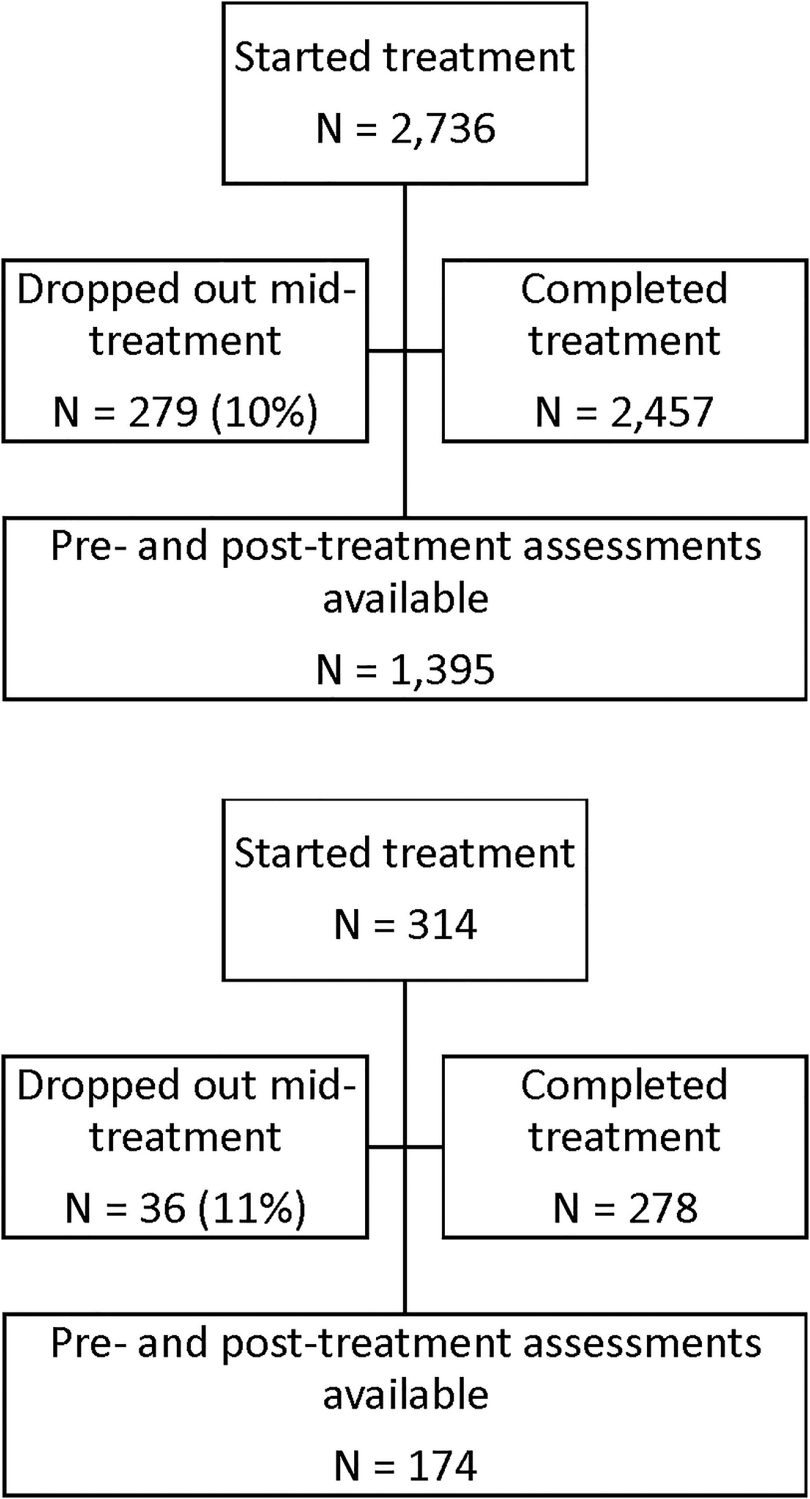
Patient flow.

A diagram of patient flow is detailed in Figure 3. For the purposes of this analysis, data were de-identified and compiled in aggregate from a secure database. At initiation of treatment, patients gave consent for inclusion in research conducted *via* analysis of de-identified data such as reported here in their acceptance of pre-treatment terms and conditions. Retrospective IRB-exempt status was granted for this analysis of de-identified data by the Institutional Review Board, University of Texas at Austin (IRB ID- STUDY00003542).

### Measures

Patient physiological metrics were uploaded *via* Wi-Fi or cellular LTE to a secure server at the completion of each session and maintained in a database. Respiratory metrics (average respiratory rate and etCO2) were captured for each of the three stages of the 17-min sessions.

Self-reported panic symptom severity was measured using the 7-item Panic Disorder Severity Scale. Self-reported PTSD symptom severity was measured using the 20-item PCL-5. Baseline measures for both PD and PTSD scales were obtained by the referring clinician or recorded during an assessment/authorization interview by a licensed healthcare professional. The post-treatment assessment of both the PDSS and the PCL-5 were administered on-screen *via* the tablet computer. Patients were classified as normocapnic (etCO_2_ ≥ 37) or hypocapnic (etCO_2_ < 37) from the average baseline-stage of the first at-home treatment sessions for purposes of examining the role of changes in respiratory characteristics over the course of treatment.

### Statistical methods

For non-dropout panic patients with both pre-and-post treatment survey scores, clinical response was defined as a 40% or greater reduction in scores on the PDSS; remission was defined as a score of five or less on the PDSS (30). For non-dropout PTSD patients, treatment response was defined as a reduction of PCL-5 score ≥ 10 points (31). Proportions of participants with the desired outcome and associated 95% lower bounds were estimated. Changes in mean scores were compared using a *t*-test and effect sizes calculated. A modified intent to treat analysis was also performed which included all patients who were trained on the treatment during the time frame in which pre- and post-treatment scales were available.

In order to more fully triangulate the construct of *clinically meaningful change,* we utilized the Jacobsen et al. (32) two-pronged statistical approach for determining clinically significant improvement for each participant. Briefly, this index first requires the assessment of whether each participant’s magnitude of pre-to-post change is statistically reliable. This step is accomplished by calculating the Reliable Change Index (RCI) for each participant. For those participants showing statistically reliable improvement (RCI = 1.96 or greater), a determination is then made as to whether the participant’s posttreatment score is closer to the distribution of scores for patients without the targeted disorder (PD or PTSD) or whether patient’s post-treatment score continues to fall within the distribution of scores for the PD and PTSD disordered groups.

Treatment adherence was calculated by determining the proportion of the 56 recommended CGRI sessions completed over the course of the study, based on objective data automatically captured to the cloud-based server. Because some patients completed more than the required number of respiratory sessions, we coded all patients who completed 56 or more sessions as 100% compliant; for all others, we calculated adherence as the number of completed sessions divided by 56. Treatment dropouts were defined as patients completing ≤6 sessions; these patients are included in the intent to treat analysis.

## Results

### Symptom severity

For the PD cohort, the mean PDSS score declined from 14.7 (sd = 5.8) at baseline to 7.2 (sd = 5.7) at post-treatment. This 7.5-point decline represents a 50% decrease, with a large effect size (Cohen’s *d* = 1.3). PDSS reduction of at least 40% was attained by 911 patients [65.3% (95% CI-62.7%–67.8%)]. Scores reflecting likely remission on the PDSS were recorded for 577 patients [41.4% (95%CI-38.7%–44.0%)]. Calculation of the Reliable Change Index classified 55.7% [95% CI-53.0%–58.3%] of participants as treatment responders. A modified intent to treat analysis of PD patients (*n* = 1,610) identified 979 patients as treatment responders [60.8% (95% CI-58.4%–63.2%)] and 609 patients as achieving remission [37.8% (95% CI-35.5%–40.3%)]. Calculation of the Reliable Change Index classified 51.9% [95% CI-49.5%–56.5%] of participants as treatment responders.

**Figure 4:**
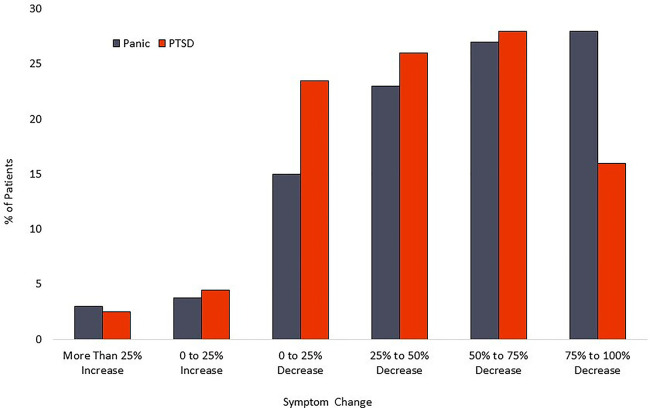
Distribution of symptom reduction.

For the PTSD cohort, the mean PCL-5 score dropped from 47.9 (sd = 15.4) at baseline to 28.2 (sd = 18.4) at posttreatment. This 19.7-point change represents a 41.1% decrease and a large effect size (Cohen’s *d* = 1.16). Within the PTSD cohort, 126 patients [72.4% (95%CI-65.0%–78.9%)] had a PCL-5 reduction of at least 10 scale points. Calculation of the Reliable Change Index classified 53.5% [95% CI- 46.0%–60.9%] of participants as treatment responders. A modified intent to treat analysis of PTSD patients (*n* = 246) recorded response rates of 56.9% [95% CI-50.5%–63.2%]. Calculation of the Reliable Change Index classified 40.6% [95%CI-34.5%–47.1%] of participants as treatment responders. Distribution of changes in symptom scores can be seen in Figure 4. Results are tabulated in Tables 2 (Completer Sample) and Table 3 (Intent to Treat Sample).

**Table 2:** Pre to post changes on primary outcomes for each diagnostic group (completer sample).

Outcome	PD (*N* = 1395)		PTSD (*N* = 174)	
	PDSS		PCL-5	
	*M*	SD	*M*	SD
Baseline	14.7	5.8	47.9	15.4
Posttreatment	7.2	5.7	28.2	18.4
*P*-Value	<0.001		<0.001	
Effect Size – Cohen’s *D*	1.31		1.16	
Reliable Change (%)	55.7		53.5	
Average Adherence (%)	74.8		74.9	

**Table 3:** Pre to post changes on primary outcomes for each diagnostic group (intent-to-treat sample).

Outcome	PD (*N* = 1610)		PTSD (*N* = 246)	
	PDSS		PCL-5	
	*M*	SD	*M*	SD
Baseline	14.7	5.7	48.8	15.0
Posttreatment	7.8	5.9	34.7	20.6
*P*-Value	<0.001		<0.001	
Effect Size – Cohen’s *D*	1.21		0.78	
Reliable Change (%)	51.9		40.6	
Average Adherence (%)	71.9		68.6	

### Adherence

**Figure 5:**
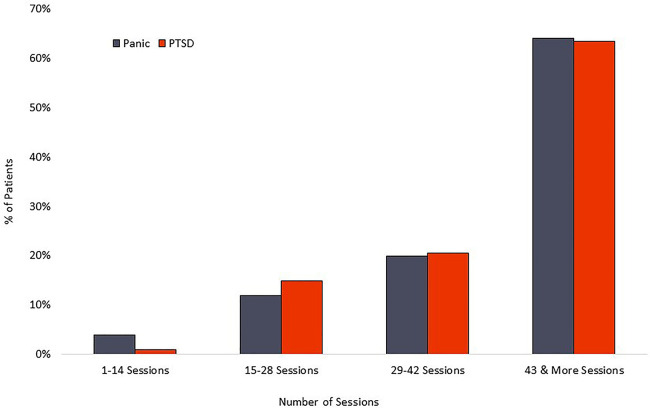
Treatment adherence.

**Figure 6:**
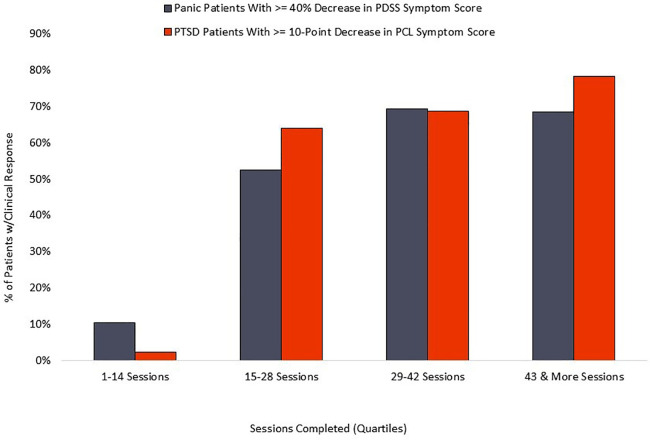
Relationship of symptom reduction to adherence.

The PD group averaged 42 completed sessions of the recommended 56, a 75% adherence rate, while the PTSD cohort averaged 42 completed sessions of the recommended 56, representing a 75% adherence rate. Distribution of overall adherence can be seen in Figure 5. The relationship between adherence and symptom reduction (as measured by percent of participants reaching clinically significant symptom reduction) is illustrated in Figure 6.

### Respiratory parameters

Of the 1,395 panic completers, 900 (65%) were classified at baseline as normocapnic and 495 (35%) were classified as hypocapnic. Pre- to post-treatment etCO_2_ mean changed from 32.8 (sd = 2.73) to 36.8 (sd = 3.96) at post-treatment for the hypocapnic group and 39.6 (sd = 2.50) to 39.9 (sd = 3.57) for the normocapnic group. The 1.15 effect size for the hypocapnic group exceeded the 0.12 value for the normocapnic group. When effect sizes for PDSS symptom reductions were calculated, the normocapnic and hypocapnic groups each showed large effect sizes (1.29 and 1.32, respectively).

Of the 174 PTSD completers, 115 (66%) were classified at baseline as normocapnic and 59 (34%) were classified as hypocapnic. Pre- to post-treatment mean etCO_2_ changed from 33.7 (sd = 2.05) to 37.2 (sd = 3.69) at post-treatment for the hypocapnic group and 39.3 (sd = 2.50) to 39.5 (sd = 3.66) for the normocapnic group. The 1.19 effect size for etCO_2_ increase in the hypocapnic group exceeded the 0.08 value for the normocapnic group. When effect sizes for PCL-5 symptom reductions were calculated, the normocapnic and hypocapnic groups each showed large effect sizes (1.12 and 1.25, respectively). Table 4 details the relationship of baseline etCO_2_ levels to outcomes.

**Table 4:** Normocapnic vs. hypocapnic subjects.

Condition	Panic Disorder		PTSD	
etCO_2_ Status	Normocapnic	Hypocapnic	Normocapnic	Hypocapnic
*N*=	900	495	115	59
Mean (sd) Baseline etCO2	39.58 (2.50)	32.84 (2.73)	39.23 (2.62)	33.66 (2.05)
Mean (sd) Final etCO2	39.94 (3.57)	36.76 (3.96)	39.49 (3.66)	37.22 (3.69)
*P* value change	0.013	<0.001	0.53	<0.001
*D* Value for etCO_2_ Change	0.112	1.15	0.083	1.193
Mean (sd) Symptom Reduction	51.1% (5.92)	48.57% (6.32)	40.3% (18.13)	42.58% (13.48)
*P* value change	<0.001	<0.001	<0.001	<0.001
*D* Value for Symptom Change	1.29	1.32	1.12	1.25

## Discussion

Our primary aim in this report is to present real-world effectiveness data for CGRI in clinical practice. Statistical analyses of outcomes in this cohort of over 1,500 patients reflect significant and clinically meaningful symptom decreases in both panic and PTSD groups, with large effect sizes when comparing mean pre-treatment to post-treatment scores. As points of reference, the clinical outcomes documented in this data set are comparable to those seen in the prior published trials. The mean 7.5-point PDSS decline compares favorably with the 9.4 recorded by Tolin et al. (20), with large effect size in each report (1.31 vs. 2.3 respectively). The 19.7-point pre-post PCL-5 decline exceeds levels accepted for clinically significant change, and the final 28.2 mean score falls at the lower range of the 28–37 cutoff scores commonly used as threshold identifying likely presence of PTSD (31). Although the PCL-5 and CAPS-5 are different instruments, network analysis methods (33) suggests that the two scales provide comparable measurement of PTSD symptoms. The large effect size for the PTSD cohort in the current analysis (1.16) approximates the 1.3 obtained in Ostacher et al. (23).

As seen in the prior CGRI trials (20, 23), patients who began treatment with hypocapnic etCO_2_ levels showed significantly greater increases in mean exhaled carbon dioxide levels following treatment than normocapnic subjects. These results conform to expectation. Normocapnic subjects would not be expected to increase these levels beyond 40 mmHg as patients are coached by the intervention to target etCO_2_ around this range value. With identical directions, the final etCO_2_ values for the hypocapnic subgroups on the other hand increased by approximately 4 mmHg, representing goal attainment and significant normalization of this respiratory measure. Prior studies have shown that both normocapnic and hypocapnic subjects achieve significant clinical benefit after use of this intervention. Similar results are seen in the present study, with large effect sizes (range 1.11 to 1.32) across the normocapnic and hypocapnic subgroups for each condition.

An RCT comparing Meuret’s CART protocol with a cognitive therapy arm (24) demonstrated significant and comparable benefit from both treatments, but concluded that etCO_2_ increases were a significant mediator of change in the CART condition but not in the cognitive therapy condition. The author concluded that changes in etCO_2_ are directly responsible for some of the symptom reduction in the respiratory therapy. It is unlikely that a single mechanism of action is responsible for the clinical benefit seen consistently with the CGRI and CART protocols, with individuals with normal and depressed etCO_2_ baseline levels appear to benefit comparably.

It is possible that lower-than-normal etCO_2_ levels during a single, brief baseline measurement is an inadequate surrogate for dysfunctional breathing in symptomatic individuals. Rapid and variable respiratory rate as well as significant decreases in etCO_2_ during the “pacing” phase of the CGRI treatment are commonly observed but not analyzed in the present study. These metrics are the subject of planned subsequent analyses. In addition, significant cardio-respiratory instability has been observed during the onset of panic attacks (34), raising the possibility that individuals learning respiratory control *via* the CART/CGRI interventions may develop skills that inhibit symptom escalation at the point of interoceptive awareness of respiratory distress or in response to triggering external events. Feinstein and colleagues’ work discussed earlier (26) also suggests that respiratory stability may function within neural networks to suppress the apnea-induced anxiety that the authors implicate in symptom surge and learned avoidance. In summary, the continuing evidence of benefit to hypocapnic as well as normocapnic individuals suggests that a single measure of low baseline etCO_2_ level does not function as a meaningful biomarker for treatment response. Planned future research will look at reactivity to CO_2_ challenge and other measures of dysfunctional breathing as well as non-respiratory potential predictive biomarkers.

The relationship between completed sessions and symptom reduction (see Figure 6) suggests a distinct “dose/response” relationship. Negligible symptom reduction is detected in patients completing fewer than 15 session, with clinical response rising robustly in the next quartile of participants and plateauing or increasing by the final quartile, marked by completion of 43 or more sessions. Standard coaching protocol is to advocate for maximum adherence, and the 75% adherence rate documented here suggests that most patients respond positively to those recommendations. We observe that some patients show rapid respiratory control and symptom reduction within the first two weeks of treatment, and it is possible that adherence in this subset may decline for patients who are experiencing rapid symptom relief and are able to stabilize breathing without twice-daily formal practice. However, the current data does not examine this potential relationship, nor do we know whether maintenance of symptom reduction beyond immediate post-treatment may be diminished in patients who fail to complete a threshold mark, suggested by the current data to be in the range of 60%–70% adherence.

The real-world outcomes reported here are appropriately compared to established treatments. For panic conditions, antidepressants and benzodiazepines are considered first line medications. Risk of abuse, side effect burden, and risk of relapse following discontinuation are commonly reported challenges with psychopharmacology (35, 36). The National Center for PTSD (37) has moved medications to a second line option for PTSD, with strong first -line recommendations for manualized trauma-focused psychotherapies. While cognitive behavioral therapies are widely considered to have the strongest evidence base for psychotherapeutic treatment of the anxiety disorders, limitations are noted in key reviews of CBT regarding response rates and tolerability (38–43). In contrast, benefits of CGRI in the context of this study include brief duration of treatment, at-home administration, clinically significant symptom reduction, and favorable adherence/dropout rates.

## Limitations

Several limitations of this effectiveness study deserve comment. First, as is the case with all non-randomized treatment effectiveness studies, threats to internal validity (e.g., selection, regression to the mean, etc.) cannot be ruled out. However, there are several features of this study that increase confidence that the symptom reduction observed in both cohorts was likely due to the CGRI intervention as opposed to extraneous factors. For instance, the low drop-out rates observed for both cohorts (<11%) were well below that observed in most psychotherapy RCT’s for panic disorder or PTSD, thus reducing the likelihood that patient attrition was biasing the treatment response rate. Moreover, both PD and PTSD tend to show a chronic clinical course without treatment (44, 45), thus helping to rule out regression to the mean or spontaneous remission as likely candidates for explaining the observed symptom reduction.

As with most effectiveness studies, inclusion criteria were relaxed and geared towards clinicians’ judgement of patient suitability for treatment rather than symptom cutoff scores or other trial enrollment criteria. This resulted in some patients scoring in the marginal range of symptom severity at pre-treatment. However, the average mean PDSS and PCL-5 scores at entry were at clinically significant levels and comparable to those reported in prior CGRI trials. These data suggest that the screening and authorization processes largely enroll patients with the intended conditions.

A third limitation of this open-label trial is the absence of an active control condition or a stringent respiratory control intervention such as false respiratory feedback. Future studies are needed to disentangle whether CGRI-induced symptom changes are mediated by changes in respiratory parameters (i.e., respiration rate and etCO_2_ levels) in addition to or as opposed to alternative putative mechanisms such as expectancy effects, desensitization to dyspnea, or change in self-efficacy for controlling symptoms. A fourth limitation of this study is the absence of extended follow-up outcome assessments, thus precluding conclusions regarding the durability of the CGRI-induced symptom changes. However, prior trials have reported sustained treatment benefit at six to twelve-month follow ups (20, 21, 23). The size of the PTSD cohort is substantially less than that for PD, which reflects the more recent FDA clearance for PTSD for this treatment. Although encouraging, additional review of outcomes for CGRI in PTSD is warranted to determine if the response rates seen in this analysis remain consistent as treatment volumes increase.

Finally, many potential prognostic variables (e.g., comorbid psychiatric and medical conditions, prior history of treatment, duration of disorder, etc.) were not included as part of data capture. Data concerning concurrent treatment with psychotherapy or medication were not obtained, thus presenting an important confound in the study, i.e., whether the benefits obtained were independent of or synergistic with other therapies.

## Challenges and treatment enhancements

While positive clinical benefit and adherence levels are described in the paper, experience and patient feedback point to certain areas needing improvement. The CGRI protocol provides the same instructions and performance targets regardless of baseline patient characteristics. As an example, a patient with significantly below-normal etCO_2_ and/or rapid, unstable respiratory rate is given the same set of instructions and targets as a patient with more normal baseline respiratory style. Perhaps as a consequence, some patients experience distressing air hunger in the early stages of treatment, as reduced respiratory volume is the behavior necessary to raise etCO_2_ in hypocapnic users. With coaching support and education, many individuals tolerate this side effect and persist, while others may discontinue treatment. A related complaint comes from individuals who are self-described “perfectionists” who are frustrated with their inability to hit the 40 mmHg target initially and express dissatisfaction or distress related to lack of perceived success. No systematic method for identifying or ameliorating these or other reasons for non-compliance is currently in place. Evaluation of CGRI in randomized control studies with sham or active control arms will be important in validating the accumulating evidence from open label and real-world trials discussed in this paper.

Future product development intends to broaden the scope of demographic and health data obtained upon registration, which may allow for greater precision in predicting which patients are likely to respond to this intervention. Additionally, gamification and individualization of the treatment protocol represents an important opportunity to improve engagement and perhaps enhance outcomes. Such efforts may optimize application to sub-populations such as adolescents and individuals with attentional difficulties.

## Conclusions

Clinically meaningful symptom reductions in both PD and PTSD patients were achieved using the CGRI treatment. The symptom reductions reported here are consistent with prior published research that tracked outcomes to six months or one year and provide encouraging evidence of clinical effectiveness when the treatment is delivered outside of a formal research setting. The embedded data analytic capacities provide automatic compilation of key outcome metrics such as those reported in this paper. Dissemination of real-world data such as these are vital for evaluating the viability (clinical benefit as well as engagement) of emerging treatments such as CGRI. Adoption of prescription digital therapeutics such as CGRI hold promise for expanding access and patient choice in the treatment of panic disorder and PTSD.

## Data Availability

The raw data supporting the conclusions of this article will be made available by the authors, without undue reservation.
